# Number of public health nurses and COVID-19 incidence rate by variant type: an ecological study of 47 prefectures in Japan

**DOI:** 10.1265/ehpm.22-00013

**Published:** 2022-05-03

**Authors:** Kimiko Tomioka, Midori Shima, Keigo Saeki

**Affiliations:** Nara Prefectural Health Research Center, Nara Medical University, Kashihara, Nara, Japan

**Keywords:** COVID-19, Incidence, Public health nurse, Ecological studies, Japan

## Abstract

**Background:**

Community health activities by public health nurses (PHNs) are known to improve lifestyle habits of local residents, and may encourage the practice of infectious disease prevention behaviors during the COVID-19 pandemic. We investigated the association between prefecture-level COVID-19 incidence rate and the number of PHNs per population in Japan, by the COVID-19 variant type.

**Methods:**

Our data were based on government surveys where prefectural-level data are accessible to the public. The outcome variable was the COVID-19 incidence rate (i.e., the cumulative number of COVID-19 cases per 100,000 population for each variant type in 47 prefectures). The explanatory variable was the number of PHNs per 100,000 population by prefecture. Covariates included socioeconomic factors, regional characteristics, healthcare resources, and health behaviors. The generalized estimating equations of the multivariable Poisson regression models were used to estimate adjusted incidence rate ratio (IRR) and 95% confidence interval (CI) for the COVID-19 cases. We performed stratified analyses by variant type (i.e., wild type, alpha variant, and delta variant).

**Results:**

A total of 1,705,224 confirmed COVID-19 cases (1351.6 per 100,000 population) in Japan were reported as of September 30, 2021. The number of PHNs per 100,000 population in Japan was 41.9. Multivariable Poisson regression models showed that a lower number of PHNs per population was associated with higher IRR of COVID-19. Among all COVID-19 cases, compared to the highest quintile group of the number of PHNs per population, the adjusted IRR of the lowest quintile group was consistently significant in the models adjusting for socioeconomic factors (IRR: 3.76, 95% CI: 2.55–5.54), regional characteristics (1.73, 1.28–2.34), healthcare resources (3.88, 2.45–6.16), and health behaviors (2.17, 1.39–3.37). These significant associations were unaffected by the variant type of COVID-19.

**Conclusion:**

We found that the COVID-19 incidence rate was higher in prefectures with fewer PHNs per population, regardless of the COVID-19 variant type. By increasing the number of PHNs, it may be possible to contain the spread of COVID-19 in Japan and provide an effective human resource to combat emerging infectious diseases in the future.

**Supplementary information:**

The online version contains supplementary material available at https://doi.org/10.1265/ehpm.22-00013.

## Introduction

Socioeconomic disparities in COVID-19 outcomes have been reported by the United States and other Western countries [1–4], followed by similar socioeconomic disparities in Asian countries including Japan [5, 6]. Specifically, morbidity and mortality due to COVID-19 are higher in low-income earners, low-wage/precarious workers, disadvantaged ethnic groups, immigrants, those who live in crowded homes, and those who live in areas with high poverty levels. Clarifying the existence of such socioeconomic disparities is important for anti-COVID-19 measures, especially in considering which population groups should be given priority for vaccination [6]. Although vaccination is an effective means for the prevention of infectious diseases [7], with regard to COVID-19, preventing the spread of infection by vaccines has not been achieved as expected [8, 9]. Therefore, in addition to general preventive measures for infectious disease, other measures to prevent COVID-19 infection among the population are required [10].

In Japan, a public health nurse is defined by the “Act on Public Health Nurses, Midwives and Nurses” as “a person who is licensed by the Minister of Health, Labour and Welfare and engages in health promotion using the name of a public health nurse” [11]. Public health nurses play a role as medical professionals who contribute to the prevention of illness and maintenance of health of many people through health promotion [11]. Public health nurses work mainly at government agencies (municipalities and public health centers) due to the nature of their work of protecting the health of individuals and communities [12]. The activities of public health nurses are wide-ranging, such as providing health consultations to residents, conducting infant health examinations, visiting the homes of residents with intractable diseases and mental disorders, and conducting educational activities on lifestyle-related diseases, infectious diseases, and addictions [13]. Previous studies in Japan reported that the large number of public health nurses per population was significantly associated with long disability-free life expectancy [14] and a high rate of gastric cancer screening [15]. An American study reported that nutritional counseling by public health nurses significantly improved the diet of rural patients with high blood cholesterol [16]. In Nagano Prefecture, Japan, it is reported that community health activities by public health nurses have increased the proportion of local residents with health-promoting behaviors, leading to an extension of the healthy life expectancy of the citizens of the prefecture [17]. These reports [14–17] suggest that the large number of public health nurses may encourage local residents to take not only actions to improve their health, but also preventive actions against COVID-19.

In many countries, including Japan, public health nurses provide essential services to protect the health of people in the community and they serve as the front line of prevention in the event of a public health crisis such as the COVID-19 pandemic [18, 19]. Although the number of public health nurses is considered to be one of the key factors in controlling the spread of COVID-19, we could not find any previous studies reporting that the high number of public health nurses per population was associated with low morbidity of COVID-19. Additionally, in previous studies targeting the Japanese population, it has been reported that the transmissibility of the alpha variant is stronger than that of the original wild-type strain [20, 21], and that the transmissibility of the delta variant is stronger than that of the alpha variant [9, 20]. Because the transmissibility of COVID-19 increases with each appearance of COVID-19 variants, strategies that were once effective in controlling the spread of COVID-19 may no longer be effective after the emergence of a new variant type [9, 20, 21]. Although we failed to find previous studies on how COVID-19 variants affect the association between the number of public health nurses and COVID-19 outcomes in Japan, we had the hypothesis that the variant type might affect the relationship between the COVID-19 incidence rate and the number of public health nurses, for example, as new variants emerged, the association would weaken.

In this study, we conducted a prefecture-wide ecological study using open data and examined the relationship between the COVID-19 incidence rate and the number of public health nurses per population, taking into account the socioeconomic factors pointed out in previous studies and the effects of the COVID-19 variants.

## Methods

The study design is a cross-sectional ecological study at prefecture level. All data used in this study consist of publicly available data by prefecture.

### Outcome variable: COVID-19 incidence rate

The Ministry of Health, Labour and Welfare updates the data on COVID-19 every day and publishes it on the website as open data [22]. Prefectural data on the number of newly confirmed cases by day after January 16, 2020 can be downloaded in the form of an Excel file. We obtained this open data on COVID-19 on November 6, 2021, and calculated the cumulative number of COVID-19 cases for each variant type by adding up the number of newly confirmed cases by prefecture within a specific period as described below.

The time of spread of each COVID-19 variant was defined with reference to previous studies in Japan [9, 20, 23, 24]. Of the five waves of the COVID-19 pandemic in Japan, the period from January 16, 2020 to the third wave is the wild type, the fourth wave is the alpha variant, and the fifth wave is the delta variant. Although the duration of the wave and the emergence of the first identified case of the COVID-19 variant differ depending on the prefecture, a uniform period has been adopted nationwide for each COVID-19 variant type based on changes in the number of new COVID-19 cases in Japan and the issuance period of the state of emergency. Therefore, in this study, we defined the wild type period as being from January 16, 2020 to the end of February 2021, the alpha variant period from March 1, 2021 to the end of May 2021, and the delta variant period from June 1, 2021 to the end of September 2021.

In calculating COVID-19 incidence rate by prefecture, the numerator used the cumulative number of COVID-19 cases for each variant type mentioned above, and the denominator used the latest available population data (October 1, 2019). Population data is updated annually by the Statistics Bureau of Japan [25], and the latest census was conducted in October 2020 [26]. However, as of November 6, 2021, when we obtained the open data for COVID-19, only data from the population as of October 1, 2019 was available. We therefore used data from the 2019 population. The original COVID-19 data (i.e., the cumulative number of COVID-19 cases and the 2019 population) are shown in Additional file 1.

A total of 1,705,224 confirmed COVID-19 cases were reported in Japan as of September 30, 2021. Of these nationwide totals, 4,382 cases were excluded from this prefectural level analysis due to unknown prefectures.

### Explanatory variable: the number of public health nurses per 100,000 population

The Report on Public Health Administration and Services conducted biennially by the Ministry of Health, Labour and Welfare [12] surveys the number of medical personnel (public health nurses, midwives, nurses, associate nurses, dental hygienists, etc.) who are currently engaged in medical settings and health services. The latest published report is 2018. The 2018 Report on Public Health Administration and Services used the “Population Estimate (as of October 1, 2018)” released by the Statistics Bureau of Japan [25] when calculating the number of medical personnel per population. In this study, we used the data on the number of public health nurses per 100,000 population by prefecture, which was reported in the 2018 Report on Public Health Administration and Services.

### Covariates

We selected the following four categories as covariates necessary for examining the relationship between the COVID-19 incidence rate and the number of public health nurses per population: socioeconomic factors, regional characteristics, healthcare resources, and health behaviors. Details such as the definition and data source of each variable are shown in Additional file 2. Because this study is an ecological study using data from 47 prefectures, the number of variables in each category was limited to four. The reasons why each variable was adopted as a covariate in this study are presented in Additional file 3.

### Statistical analysis

To investigate the relationship between the COVID-19 incidence rate and the number of public health nurses across 47 prefectures, we used the generalized estimating equations of the multivariable Poisson regression model and calculated an incidence rate ratio (IRR) and a 95% confidence interval (CI) for COVID-19 cases. The independent variable was the number of public health nurses per 100,000 population in each prefecture. The 47 prefectures were classified into quintile groups according to their number of public health nurses per population, and the 5th quintile group with the highest number of public health nurses per population was designated as the reference group. We set the logarithm of the cumulative number of the COVID-19 cases for each prefecture as the outcome variable, and included the logarithm of the population by prefecture as an offset term. The base of the logarithmic conversion was e. Based on the histogram, the cumulative number of COVID-19 cases was approximate to the Poisson distribution, and Pearson residual plot of the model showed appropriate distribution.

First, we calculated a crude IRR for the COVID-19 cases. Next, in Model 1, we adjusted for the 4 socioeconomic factors (household income, Gini coefficient, proportion of unemployed people, and proportion of protected persons). In Model 2, we adjusted for the 4 regional characteristics (proportion of people aged ≥65, proportion of tertiary industry workers, household crowding, and annual mean temperature). In Model 3, we adjusted for the 4 healthcare resources (number of physicians, nurses, civil servants, and acute care hospital beds per population). In the final Model 4, we adjusted for the 4 health behaviors (health checkup prevalence, volunteer activity participation prevalence, smoking prevalence, and obesity prevalence). There was no multicollinearity among covariates with a variance inflation factor over 2.

The analyses were performed according to the variant type of COVID-19 to examine whether the association between the COVID-19 incidence rate and the number of public health nurses was affected by the variant type of COVID-19.

Statistical analyses were performed using the IBM SPSS Statistics Ver. 27 for Windows (Armonk, New York, US), and a significant level was set at 0.05 (two-tailed test).

## Results

**Fig. 1:**
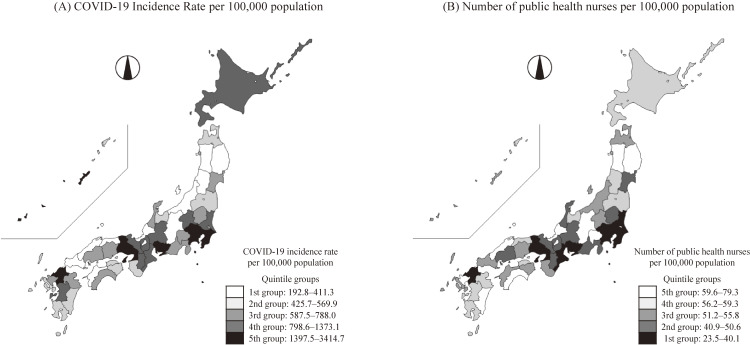
Color-coded map of COVID-19 incidence rate and the number of PHNs in 47 Prefectures, Japan. PHNs; public health nurses. For the color settings, black is high risk and white is low risk. That is, high risk (i.e., black) means that the COVID-19 incidence rate is high and the number of public health nurses is small. COVID-19 incidence rate is based on all COVID-19 cases from January 16, 2020 to the end of September 2021. The number of public health nurses per 100,000 population is based on the 2018 survey data.

Regarding the COVID-19 incidence rate per 100,000 population in Japan, the wild type period was 343.0, the alpha variant period was 248.8, the delta variant period was 759.8, and all cases period was 1351.6. The number of public health nurses per 100,000 population in Japan was 41.9. The number of public health nurses per 100,000 population for each quintile group was 23.5–40.1 in the lowest quintile group, 40.9–50.6 in the second quintile group, 51.2–55.8 in the third quintile group, 56.2–59.3 in the fourth quintile group, and 59.6–79.3 in the highest quintile group. The COVID-19 incidence rate by prefecture and variant type and the number of public health nurses per 100,000 population by prefecture are presented in Additional file 1. The COVID-19 incidence rate and the number of public health nurses showed similar distribution in color-coded maps (Fig. 1).

Among all COVID-19 cases, there was a consistent pattern: Higher IRRs were found in prefectures with the smallest number of public health nurses per population (Table 1). Compared to the 5th quintile group, the adjusted IRR (95% CI) of the 1st quintile group was 3.76 (2.55–5.54) after adjusting for socioeconomic factors (Model 1), 1.73 (1.28–2.34) after adjusting for regional characteristics (Model 2), 3.88 (2.45–6.16) after adjusting for healthcare resources (Model 3), and 2.17 (1.39–3.37) after adjusting for health behaviors (Model 4). Additionally, *P* for trend was less than 0.001 in Model 1, Model 3, and Model 4, and 0.065 in Model 2. After stratified analyses by variant type, the adjusted IRR of the 1st quintile group was not significant only for the alpha variant in Model 4 (IRR = 1.69, 95% CI = 0.96–2.97, *P*-value = 0.071), but all others showed statistically significant results with higher IRR: The minimum is the delta variant in Model 2 (IRR = 1.55, 95% CI = 1.23–1.96, *P*-value <0.001), and the maximum is the wild type in Model 1 (IRR = 4.46, 95% CI = 2.71–7.34, *P*-value <0.001).

**Table 1:** Association between COVID-19 incidence rate and number of PHNs per population, by variant type

**Number of PHNs ** **per population**	**Wild type**		**Alpha variant**		**Delta variant**		**All cases**	
	**IRR^a^ (95% CI)**	***P***	**IRR^a^ (95% CI)**	***P***	**IRR^a^ (95% CI)**	***P***	**IRR^a^ (95% CI)**	***P***
Crude Model								
4th quintile	1.96 (0.99–3.88)	0.053	2.20 (1.22–3.96)	0.009	1.38 (0.99–1.92)	0.056	1.68 (1.08–2.61)	0.021
3rd quintile	1.66 (0.81–3.37)	0.164	2.22 (1.26–3.90)	0.006	2.03 (0.87–4.72)	0.101	1.99 (0.96–4.12)	0.065
2nd quintile	2.05 (1.38–3.04)	<0.001	2.02 (1.41–2.90)	<0.001	1.98 (1.45–2.70)	<0.001	2.00 (1.51–2.65)	<0.001
1st quintile	5.05 (3.28–7.77)	<0.001	3.41 (2.29–5.09)	<0.001	4.26 (3.01–6.02)	<0.001	4.26 (3.10–5.85)	<0.001
*P* for trend	<0.001		0.011		<0.001		<0.001	
Model 1^b^								
4th quintile	1.73 (1.00–2.97)	0.049	1.74 (0.98–3.06)	0.058	1.27 (0.94–1.71)	0.118	1.48 (1.03–2.13)	0.035
3rd quintile	1.34 (0.77–2.32)	0.301	1.55 (0.96–2.51)	0.072	1.67 (0.94–2.96)	0.078	1.57 (0.97–2.54)	0.064
2nd quintile	2.36 (1.54–3.63)	<0.001	2.27 (1.36–3.76)	0.002	2.22 (1.63–3.03)	<0.001	2.26 (1.66–3.08)	<0.001
1st quintile	4.46 (2.71–7.34)	<0.001	2.88 (1.72–4.85)	<0.001	3.84 (2.47–5.98)	<0.001	3.76 (2.55–5.54)	<0.001
*P* for trend	<0.001		<0.001		<0.001		<0.001	
Model 2^c^								
4th quintile	1.46 (0.93–2.30)	0.099	1.94 (1.13–3.33)	0.016	1.16 (0.86–1.57)	0.324	1.39 (0.96–2.00)	0.081
3rd quintile	1.21 (0.80–1.84)	0.374	1.96 (1.29–2.97)	0.001	1.20 (0.94–1.54)	0.145	1.36 (1.05–1.76)	0.020
2nd quintile	1.42 (0.95–2.13)	0.085	1.83 (1.08–3.09)	0.025	1.31 (1.00–1.71)	0.049	1.41 (1.08–1.85)	0.012
1st quintile	1.84 (1.28–2.65)	0.001	2.35 (1.04–5.28)	0.039	1.55 (1.23–1.96)	<0.001	1.73 (1.28–2.34)	<0.001
*P* for trend	0.013		0.363		0.003		0.065	
Model 3^d^								
4th quintile	2.04 (1.07–3.88)	0.031	1.72 (1.07–2.79)	0.026	1.54 (1.06–2.25)	0.025	1.73 (1.12–2.67)	0.013
3rd quintile	1.58 (0.78–3.17)	0.201	2.25 (1.05–4.86)	0.038	1.89 (0.97–3.68)	0.060	1.92 (0.98–3.75)	0.058
2nd quintile	1.95 (1.18–3.23)	0.009	2.30 (1.25–4.25)	0.008	1.98 (1.28–3.06)	0.002	2.04 (1.32–3.13)	0.001
1st quintile	4.14 (2.40–7.14)	<0.001	4.30 (2.22–8.30)	<0.001	3.58 (2.26–5.68)	<0.001	3.88 (2.45–6.16)	<0.001
*P* for trend	<0.001		<0.001		<0.001		<0.001	
Model 4^e^								
4th quintile	1.49 (0.86–2.58)	0.155	1.40 (0.88–2.23)	0.155	1.06 (0.72–1.56)	0.777	1.24 (0.84–1.83)	0.279
3rd quintile	0.93 (0.49–1.80)	0.836	1.23 (0.70–2.16)	0.470	1.10 (0.70–1.73)	0.686	1.09 (0.68–1.74)	0.720
2nd quintile	1.51 (0.94–2.44)	0.092	1.43 (0.86–2.40)	0.171	1.58 (1.02–2.45)	0.042	1.53 (1.04–2.25)	0.030
1st quintile	2.25 (1.30–3.89)	0.004	1.69 (0.96–2.97)	0.071	2.37 (1.49–3.77)	<0.001	2.17 (1.39–3.37)	0.001
*P* for trend	0.009		0.140		<0.001		<0.001	

CI, confidence interval; IRR, incidence rate ratio; PHNs, public health nurses.

Reference: the 5th quintile group of the number of public health nurses per population.

^a^Incidence rate ratio was calculated using Poisson regression models with log (population) as the offset.

^b^Model 1 was adjusted for socioeconomic factors (i.e., household income, Gini coefficient, proportion of unemployed people, and proportion of protected persons). ^c^Model 2 was adjusted for regional characteristics (i.e., proportion of people aged ≥65, proportion of tertiary industry workers, household crowding, and annual mean temperature). ^d^Model 3 was adjusted for healthcare resources (i.e., the number of physicians, nurses, civil servants, and acute care hospital beds per population). ^e^Model 4 was adjusted for health behaviors (i.e., health checkup prevalence, volunteer activity participation prevalence, smoking prevalence, and obesity prevalence).

Regarding Model 2, initially, we listed not only the proportion of people aged ≥65, but also the population density as one of the important variables for regional characteristics. However, we were unable to put these two variables into the covariates at the same time due to the high correlation between the proportion aged ≥65 and the population density, which raises the issue of multicollinearity. An additional analysis was performed in Model 2 (Model 2A), where the proportion of people aged ≥65 was replaced by population density (Additional file 4). As a result, similar results were obtained.

## Discussion

This ecological study investigated the cross-sectional association between the prefecture-level COVID-19 incidence rate and the number of public health nurses per population in Japan, by the variant type of COVID-19. We found that the COVID-19 incidence rate was higher in prefectures with fewer public health nurses per population, regardless of the COVID-19 variant type. To our knowledge, this study was the first to suggest that increasing the number of public health nurses in prefectures is effective as a measure to prevent the spread of COVID-19 in Japan.

Although the mechanism of the relationship between the COVID-19 incidence rate and the number of public health nurses is unclear, the following may provide a possible explanation. First, the number of COVID-19 cases and deaths in Japan is one of the lowest in the world [27, 28], which may be due to the role played by public health centers in Japan. Public health centers work within guidelines set by the Community Health Act [29], and in the response to the COVID-19 pandemic, it has mainly been public health nurses who investigate the behavior history of COVID-19 patients before the onset of the disease to identify the source of infection and actively track down close contacts [30]. This approach has been described as a cluster-based approach or retrospective contact tracing, contributing to the low number of cases in Japan [30, 31]. Second, European studies have reported lower numbers of COVID-19 cases and deaths in countries with high social capital [32]. A Japanese study also reported that the number of COVID-19 deaths was low in prefectures with high social capital [33]. Previous studies [32, 33] suggest that social capital leads to compliance with COVID-19-related policies and practice of COVID-19 prevention actions. Nagano Prefecture reported that the development of community health prevention activities by public health nurses led to active participation in volunteer activities [17]. Because volunteer activity participation prevalence in the community is used as a component of the social capital index in Japan [34], community health activities by public health nurses may lead to the growth of local social capital. Therefore, residents living in prefectures with larger numbers of public health nurses tend to adhere more to COVID-19 preventive measures such as mask-wearing, vaccination, and refraining from going out, which may help prevent the spread of COVID-19. The initial hypothesis assumed the effect of COVID-19 variants, but the results of this study showed that the variant type had no effect. The reason for this may be that the assumed mechanisms (i.e., retrospective contact tracing conducted by public health nurses and maturation of social capital through community health activities by public health nurses) are not easily affected by the transmissibility of the variant strain. Therefore, it is considered that the relationship between the COVID-19 incidence rate and the number of public health nurses per population can be seen regardless of the COVID-19 variants.

Our study has some limitations. First, this is an ecological study examining the relationship between the COVID-19 incidence rate and the number of public health nurses in regional populations. Due to ecological fallacy, the association observed in this study may not apply at the individual level. Moreover, it is difficult to establish a causal relationship because this study has a cross-sectional design. Second, the data on the number of public health nurses used in this study is from 2018, not from the period of the COVID-19 pandemic. It is highly possible that the number of public health nurses has been increased in the COVID-19 crisis [35]. However, one of our hypotheses is that community health activities by public health nurses during normal times promote the practice of infectious disease preventive actions during the COVID-19 pandemic. Therefore, using data on the number of public health nurses in 2018 before the COVID-19 pandemic would not be a serious problem in this study. Third, the COVID-19 data released by the Ministry of Health, Labour and Welfare only includes the number of infected people on a daily basis at the prefecture level, and the gender and age are unknown. There are prefectural differences in the male-to-female ratio and the ratio of older people [25, 26]. In particular, the percentage of people aged 65 and older varies widely from 22.2% to 37.2%. It should be noted that the results of this study are based on the raw COVID-19 incidence rate, which is adjusted for neither gender nor age. Fourth, the number of public health nurses used in this study was the total number. We could not analyze what kind of workplace the public health nurses were engaged in. According to the Report on Public Health Administration and Services [12], 73.9% of working public health nurses are engaged in community health services at public health centers and municipalities, and the rest work in business offices (6.3%), hospitals (6.2%), clinics (3.8%), and long-term care insurance facilities (2.5%), etc. Regarding anti-COVID-19 measures, public health nurses at public health centers are engaged in public health interventions based on retrospective contact tracing [30], and municipal public health nurses are engaged in vaccination [36]. Regarding community health activities by public health nurses in normal times, municipalities provide health services that directly interact with residents, and public health centers provide the necessary technical assistance to health services provided by municipalities [37, 38]. Our findings show that the number of public health nurses per population contributes to the low number of COVID-19 cases. However, because the activities of public health nurses differ depending on the place of employment, we have not been able to confirm in which sector an increase in the number of public health nurses would be effective in combating COVID-19. Finally, in this study, the main confounding factors could be adjusted. However, because the sample size is the number of prefectures (that is, 47), our study was limited in the covariate information that could be incorporated into the model. For example, various measures of local governments such as health administration and economic policy cannot be adjusted in this analysis. Therefore, there is a possibility of residual confounders in this study.

Although there are limitations to this study, our study also has some strengths. First, because the open data used in this study is prefectural data based on large-scale national surveys, it has the advantages of high representativeness and high reliability. Second, our study used a wide range of confounding factors as covariates that are important in the association between the COVID-19 incidence rate and the number of public health nurses. Finally, this study shows that increasing the number of public health nurses, which has received little attention in previous studies, provides an effective human resource to prevent the spread of COVID-19. Regarding the generalizability of this study, the work content and lifelong education system of public health nurses differ from country to country [18, 38, 39]. In addition, the infection status of COVID-19 also differs from country to country [28]. Japan has a relatively solid education system for public health nurses, and has a relatively low infection rate in comparison to the rest of the world. Therefore, it is necessary to be cautious about whether the association between the COVID-19 incidence rate and the number of public health nurses found in our Japanese ecological study can be generalized is also relevant to other countries. Further ecological studies would need to be carried out in other countries in order to confirm the results of this study.

Regarding the direction of future research, we are considering two research projects. First, since 2020, many prefectures have been increasing the number of public health nurses for the purpose of strengthening the functions of their COVID-19 response [35, 40]. In future studies, therefore, it will be important to examine whether the spread of COVID-19 may be prevented by increasing the number of public health nurses. Second, in Japan, vaccinations started on May 3, 2021 [41]. As of the end of September 2021, the final day of the observation period in this study, the proportion of people who were fully vaccinated was 57.4% of the population [41]. Future studies should therefore examine whether the association between the morbidity from new COVID-19 variants and the number of public health nurses is affected by the proportion of fully vaccinated people at the prefectural level.

In conclusion, our results suggest that increasing the number of public health nurses may help to contain the spread of COVID-19 in Japan, and that the efficacy of this measure may be less susceptible to the variants of COVID-19. Policy makers should consider measures to increase the number of public health nurses, recognizing that public health nurses will be an important human resource for pandemics of newly emerging infectious diseases such as COVID-19.

## Abbreviations

CI: confidence interval
IRR: incidence rate ratio

## Supplementary information

**Additional file 1:** COVID-19 data on the cumulative number of cases and the incidence rate by prefecture and variant type, and the number of public health nurses per population by prefecture.

**Additional file 2:** Definition and data source of covariates.

**Additional file 3:** Reasons for adoption as a covariate.

**Additional file 4:** Association between COVID-19 incidence rate and number of PHNs per population, by variant type: Additional analysis for Model 2.

## Declarations

### Availability of data and materials

All data used in this study are publicly available. Data can be downloaded from following websites: the number of COVID-19 cases (https://www.mhlw.go.jp/stf/covid-19/kokunainohasseijoukyou.html) and the number of public health nurses (https://www.mhlw.go.jp/toukei/list/36-19.html). Details of the data source of each variable adopted as a covariate in this study are presented in Additional file 2.
