# Supplementary material for: Number of public health nurses and COVID-19 incidence rate by variant type: an ecological study of 47 prefectures in Japan
Source: Environ Health Prev Med. 2022 May 3;27:18. doi: 10.1265/ehpm.22-00013 (PMC9251616; doi:10.1265/ehpm.22-00013)
Supplement: Supplementary file 1 — Additional file 1: COVID-19 data on the cumulative number of cases and the incidence rate by prefecture and variant type, and the number of public health nurses per population by prefecture. [file ehpm-27-018-s001.pdf]

Additional file 1. COVID-19 data on the cumulative number of cases and the incidence rate by prefecture and variant type, and the number of public health nurses per population by prefecture

		Cumulative number of COVID-19 cases[a]				Population	Incidence rate per 100,000 population				Explanatory variable
		Wild type[b]	Alpha variant[c]	Delta variant[d]	All cases[e]	(thousand persons)	Wild type[b]	Alpha variant[c]	Delta variant[d]	All cases[e]	Number of PHNs per 100,000 population[f]
Overall		432,791	313,851	958,582	1,705,224	126,167	343.0	248.8	759.8	1351.6	41.9
Prefectures											
1	Hokkaido	19,093	18,866	22,279	60,238	5,250	363.7	359.4	424.4	1147.4	59.3
2	Aomori	816	1,536	3,340	5,692	1,246	65.5	123.3	268.1	456.8	54.2
3	Iwate	554	888	2,037	3,479	1,227	45.2	72.4	166.0	283.5	60.0
4	Miyagi	3,623	5,231	7,350	16,204	2,306	157.1	226.8	318.7	702.7	47.5
5	Akita	269	486	1,107	1,862	966	27.8	50.3	114.6	192.8	59.6
6	Yamagata	543	1,418	1,537	3,498	1,078	50.4	131.5	142.6	324.5	57.2
7	Fukushima	1,947	2,636	4,856	9,439	1,846	105.5	142.8	263.1	511.3	56.9
8	Ibaraki	5,755	3,979	14,406	24,140	2,860	201.2	139.1	503.7	844.1	40.1
9	Tochigi	4,093	2,249	8,898	15,240	1,934	211.6	116.3	460.1	788.0	49.0
10	Gumma	4,502	3,319	8,745	16,566	1,942	231.8	170.9	450.3	853.0	51.2
11	Saitama	29,342	14,431	70,893	114,666	7,350	399.2	196.3	964.5	1560.1	30.3
12	Chiba	26,458	10,673	62,400	99,531	6,259	422.7	170.5	997.0	1590.2	33.3
13	Tokyo	111,676	49,256	218,547	379,479	13,921	802.2	353.8	1569.9	2725.9	28.4
14	Kanagawa	44,898	16,617	106,074	167,589	9,198	488.1	180.7	1153.2	1822.0	23.5

Additional file 1. Continued.

		Cumulative number of COVID-19 cases[a]				Population	Incidence rate per 100,000 population				Explanatory variable
		Wild type[b]	Alpha variant[c]	Delta variant[d]	All cases[e]	(thousand persons)	Wild type[b]	Alpha variant[c]	Delta variant[d]	All cases[e]	Number of PHNs per 100,000 population[f]
15	Niigata	1,080	2,126	4,634	7,840	2,223	48.6	95.6	208.5	352.7	54.3
16	Toyama	905	964	2,924	4,793	1,044	86.7	92.3	280.1	459.1	59.1
17	Ishikawa	1,851	1,901	4,094	7,846	1,138	162.7	167.0	359.8	689.5	49.7
18	Fukui	545	495	2,005	3,045	768	71.0	64.5	261.1	396.5	61.6
19	Yamanashi	939	611	3,564	5,114	811	115.8	75.3	439.5	630.6	76.5
20	Nagano	2,364	2,425	3,934	8,723	2,049	115.4	118.4	192.0	425.7	77.2
21	Gifu	4,616	3,998	9,775	18,389	1,987	232.3	201.2	491.9	925.5	49.5
22	Shizuoka	5,141	3,249	18,210	26,600	3,644	141.1	89.2	499.7	730.0	47.0
23	Aichi	25,889	21,400	58,252	105,541	7,552	342.8	283.4	771.3	1397.5	36.2
24	Mie	2,528	2,385	9,704	14,617	1,781	141.9	133.9	544.9	820.7	40.9
25	Shiga	2,467	2,640	7,132	12,239	1,414	174.5	186.7	504.4	865.6	50.6
26	Kyoto	9,063	6,756	19,647	35,466	2,583	350.9	261.6	760.6	1373.1	45.8
27	Osaka	47,121	52,585	99,844	199,550	8,809	534.9	596.9	1133.4	2265.3	25.9
28	Hyogo	17,968	21,727	37,633	77,328	5,466	328.7	397.5	688.5	1414.7	32.1
29	Nara	3,283	4,484	7,602	15,369	1,330	246.8	337.1	571.6	1155.6	41.0
30	Wakayama	1,163	1,455	2,617	5,235	925	125.7	157.3	282.9	565.9	52.9
31	Tottori	210	255	1,172	1,637	556	37.8	45.9	210.8	294.4	59.3
32	Shimane	284	256	1,073	1,613	674	42.1	38.0	159.2	239.3	79.3
33	Okayama	2,484	4,856	7,754	15,094	1,890	131.4	256.9	410.3	798.6	53.6
34	Hiroshima	5,025	5,663	10,937	21,625	2,804	179.2	202.0	390.0	771.2	46.1
35	Yamaguchi	1,382	1,542	2,662	5,586	1,358	101.8	113.5	196.0	411.3	55.5

Additional file 1. Continued.

		Cumulative number of COVID-19 cases[a]				Population	Incidence rate per 100,000 population				Explanatory variable
		Wild type[b]	Alpha variant[c]	Delta variant[d]	All cases[e]	(thousand persons)	Wild type[b]	Alpha variant[c]	Delta variant[d]	All cases[e]	Number of PHNs per 100,000 population[f]
36	Tokushima	452	1,179	1,622	3,253	728	62.1	162.0	222.8	446.8	55.8
37	Kagawa	750	1,252	2,677	4,679	956	78.5	131.0	280.0	489.4	58.5
38	Ehime	1,063	1,650	2,436	5,149	1,339	79.4	123.2	181.9	384.5	51.9
39	Kochi	884	584	2,633	4,101	698	126.6	83.7	377.2	587.5	73.9
40	Fukuoka	18,061	16,033	39,817	73,911	5,104	353.9	314.1	780.1	1448.1	35.8
41	Saga	1,057	1,446	3,244	5,747	815	129.7	177.4	398.0	705.2	60.2
42	Nagasaki	1,612	1,280	3,091	5,983	1,327	121.5	96.5	232.9	450.9	56.2
43	Kumamoto	3,443	2,806	8,028	14,277	1,748	197.0	160.5	459.3	816.8	56.5
44	Oita	1,293	2,076	4,719	8,088	1,135	113.9	182.9	415.8	712.6	58.7
45	Miyazaki	1,947	1,078	3,090	6,115	1,073	181.5	100.5	288.0	569.9	62.3
46	Kagoshima	1,761	1,566	5,724	9,051	1,602	109.9	97.8	357.3	565.0	58.3
47	Okinawa	8,193	8,813	32,609	49,615	1,453	563.9	606.5	2244.3	3414.7	53.0

PHNs, public health nurses.

[a] Data source is COVID-19 open data released by the Ministry of Health, Labour and Welfare, Japan.

[b] The wild type period is from January 16, 2020 to the end of February 2021.

[c] The Alpha variant period is from March 1, 2021 to the end of May 2021.

[d] The Delta variant period is from June 1, 2021 to the end of September 2021.

[e] All cases period is from January 16, 2020 to the end of September 2021.

[f] Data source is the Report on Public Health Administration and Services 2018.
