# Supplementary material for: Number of public health nurses and COVID-19 incidence rate by variant type: an ecological study of 47 prefectures in Japan
Source: Environ Health Prev Med. 2022 May 3;27:18. doi: 10.1265/ehpm.22-00013 (PMC9251616; doi:10.1265/ehpm.22-00013)
Supplement: Supplementary file 2 — Additional file 2: Definition and data source of covariates. [file ehpm-27-018-s002.pdf]

Additional file 2. Definition and data source of covariates

Category	Variable	Definition	Data source	Implementing body	Year
Socioeconomic factors	Household income	Equivalized household disposable income adjusted by regional price parities (100,000 yen per year)	National Survey of Family Income, Consumption and Wealth	Ministry of Internal Affairs and Communications	2019
	Gini coefficient	Gini coefficient of equivalized yearly disposable income	National Survey of Family Income, Consumption and Wealth	Ministry of Internal Affairs and Communications	2019
	Proportion of unemployed people	Percentage of unemployed people in the labor force based on model estimates	Labor Force Survey	Ministry of Internal Affairs and Communications	2019
	Proportion of protected persons	Percentage of the population receiving public assistance per 100 population	National Survey on Public Assistance Recipients	Ministry of Health, Labour and Welfare	2019
Regional characteristics	Proportion of people aged ≥65	Percentage of people aged 65 and over in the total population	Population Estimates	Ministry of Internal Affairs and Communications	2019
	Proportion of tertiary industry workers	Percentage of workers employed in tertiary industry among total employed workers	National Population Census	Ministry of Internal Affairs and Communications	2015
	Household crowding	Number of tatami units of dwelling houses per person (one tatami unit means 1.65 square meters)	Housing and Land Survey	Ministry of Internal Affairs and Communications	2018
	Annual mean temperature	Yearly average of air temperature	Past Weather Data	Japan Meteorological Agency	2019

Additional file 2. Continued.

Category	Variable	Definition	Data source	Implementing body	Year
Healthcare resources	Number of physicians per population	Number of physicians working at medical facilities per 100,000 population	Statistics of Physicians, Dentists and Pharmacists	Ministry of Health, Labour and Welfare	2018
	Number of nurses per population	Number of nurses and assistant nurses working at medical facilities per 100,000 population	Report on Public Health Administration and Services	Ministry of Health, Labour and Welfare	2018
	Number of civil servants per population	Number of civil servants per 100,000 population	Employment Structure Basic Survey	Ministry of Internal Affairs and Communications	2017
	Number of acute care hospital beds per population	Number of acute care hospital beds per 1,000 population	Hospital Bed Function Report	Ministry of Health, Labour and Welfare	2018
Health behaviors	Health checkup prevalence	Age-standardized prevalence of persons having an annual medical checkup among people aged ≥20 years	Comprehensive Survey of Living Conditions	Ministry of Health, Labour and Welfare	2019
	Volunteer activity participation prevalence	Percentage of persons participating in volunteer activities among people at least 10 years old	Social Life Basic Survey	Ministry of Internal Affairs and Communications	2016
	Smoking prevalence	Age-standardized smoking prevalence among people at least 20 years old	Comprehensive Survey of Living Conditions	Ministry of Health, Labour and Welfare	2019
	Obesity prevalence	Percentage of persons whose BMI is 25 or above among people aged 40 to 74 years	NDB Open Data Japan	Ministry of Health, Labour and Welfare	2018
