# Supplementary material for: Number of public health nurses and COVID-19 incidence rate by variant type: an ecological study of 47 prefectures in Japan
Source: Environ Health Prev Med. 2022 May 3;27:18. doi: 10.1265/ehpm.22-00013 (PMC9251616; doi:10.1265/ehpm.22-00013)
Supplement: Supplementary file 3 — Additional file 3: Reasons for adoption as a covariate. [file ehpm-27-018-s003.pdf]

Additional file 3. Reasons for adoption as a covariate

For socioeconomic factors, we adopted household income, Gini coefficient, the proportion of unemployed people, and the proportion of protected persons, because they were significant explanatory variables in a previous study that reported socioeconomic disparities in the COVID-19 incidence rate in Japan [1]. The previous study [1] used mean annual household income adjusted by regional price parities, Gini coefficient based on household income, and the non-adjusted proportion of unemployed people. However, in this study, equivalized household disposable income adjusted by regional price parities, Gini coefficient of equivalized yearly disposable income, and the proportion of unemployed people based on model estimates were used as an index more suitable for the prefectural socioeconomic characteristics.

For regional characteristics, we adopted the proportion of people aged ≥65, the proportion of tertiary industry workers, household crowding, and annual mean temperature. The reasons for choosing these four variables as regional characteristics are as follows. First, we initially listed not only the proportion of people aged ≥65 but also the population density as potential variables for regional characteristics [1]. However, because Pearson's correlation coefficient between the proportion of people aged ≥65 and the logarithm of population density was as high as minus 0.80, we abandoned using both

as covariates to avoid the problem of multicollinearity. Since age is an important factor in the onset and severity of COVID-19 [2], we adopted the proportion of people aged ≥65 as one of the regional characteristics in this study. Second, the previous study [1] reported percentages of retail, transportation postal, and restaurant industry workers as significant explanatory variables. In this study, persons in these occupations were defined as tertiary industrial workers, and the proportion of tertiary industrial workers among total employed workers was used as a covariate. Third, the previous study [1] reported that the prefectural-level household crowding was a significant living environment for COVID-19 cases. Therefore, in this study, we adopted household crowding as one of the regional characteristics. Fourth, previous research suggests that the local environmental temperature is associated with an increase in newly confirmed COVID-19 cases [3, 4]. Therefore, in this study, prefectural-level annual mean temperature was selected as one of the regional characteristics.

For healthcare resources, we adopted the number of physicians, nurses, civil servants, and acute care hospital beds per population. A previous study used the number of physicians, nurses, and hospital beds per 1000 people as healthcare capacity associated with international variation in COVID-19 outcomes [5]. The reasons for adding the number of civil servants in this study are as follows. In Japan, vaccinations for COVID-

19 are carried out by municipalities under the direction of the national government [6]. In addition, public health centers are in charge of dealing with COVID-19 patients [7], but in order to reduce the burden of COVID-19-related work at public health centers, prefectures coordinate the hospitalization of patients, support housebound patients, and dispatch local government officials to public health centers [8]. Therefore, the number of civil servants in prefectures is considered to be one of the healthcare resources associated with COVID-19 outcomes in Japan.

For health behaviors, we adopted health checkup prevalence, volunteer activity participation prevalence, smoking prevalence, and obesity prevalence. For health checkup prevalence, a previous study found that those who received health examinations had better health knowledge than those without health examinations [9]. Because of a positive association between health knowledge and health-promoting behaviors [10], prefectural-level health checkup prevalence may influence the practice of infectious disease prevention behavior. For volunteer activity participation prevalence, it has been reported that the high participation prevalence of volunteer activities in the community is associated with the high prevalence of community residents with healthy behaviors [11, 12]. Therefore, volunteer activity participation prevalence is considered to be one of the indicators of health behaviors in the community. For smoking prevalence and obesity

prevalence, smokers and obese people have been identified as a group at risk of infection and more severe outcomes of communicable diseases [13]. Moreover, because health checkup prevalence is lower for younger people and smoking prevalence is lower for the older adult population [14], these two variables are affected by the proportion of people aged ≥65 in the region. Therefore, health checkup prevalence and smoking prevalence were standardized by age using the age-specific data of the Comprehensive Survey of Living Conditions [14], which is a nationwide survey. We performed direct age standardization using the 1985 model population as the reference group. The volunteer activity participation prevalence was a crude prevalence because age-specific data were not available. We also used the crude prevalence for obesity prevalence because the NDB Open Data used was limited to ages 40 to 74 years.

References

1. Yoshikawa Y, Kawachi I. Association of Socioeconomic Characteristics With Disparities in COVID-19 Outcomes in Japan. JAMA Netw Open. 2021;4:e2117060.
2. Mertoglu C, Huyut MT, Olmez H, Tosun M, Kantarci M, Coban TA. COVID-19 is more dangerous for older people and its severity is increasing: a case-control study. Med Gas Res. 2022;12:51–54.
3. Ganslmeier M, Furceri D, Ostry JD. The impact of weather on COVID-19 pandemic. Sci Rep. 2021;11:22027.
4. Hu CY, Xiao LS, Zhu HB, Zhu H, Liu L. Correlation Between Local Air Temperature and the COVID-19 Pandemic in Hubei, China. Front Public Health. 2021;8:604870.
5. Kim J, Hong K, Yum S, et al. Factors associated with the difference between the incidence and case-fatality ratio of coronavirus disease 2019 by country. Sci Rep. 2021;11:18938.
6. Ministry of Health, Labour and Welfare, Japan. Notifications, office communication, etc. for local governments regarding the COVID-19 vaccine. https://www.mhlw.go.jp/stf/seisakunitsuite/bunya/vaccine_notifications.html. Accessed 5 Mar 2022. (Japanese).
7. Yoshioka-Maeda K. Developing sustainable public health care systems for

responding to COVID-19 in Japan. Public Health Nurs. 2021;38:470–472.

8. Ministry of Health, Labour and Welfare COVID-19 Control Promotion Headquarters. Regarding the establishment of a health center system for COVID-19 in preparation for the spread of infection in the future. Office contact dated October 1, 2021. (Japanese).

9. Huang HT, Kuo YM, Wang SR, Wang CF, Tsai CH. Structural Factors Affecting Health Examination Behavioral Intention. Int J Environ Res Public Health. 2016;13:395.

10. Zhang X, Zhu M, Dib HH, Hu J, Tang S, Zhong T, Ming X. Knowledge, awareness, behavior (KAB) and control of hypertension among urban elderly in western China. Int J Cardiol. 2009;137:9–15.

11. Imamura H, Murakami Y, Okamura T, Nishiwaki Y. Relationship between health promotion volunteer experience and medical costs: Hoken-hodouin activities in Suzaka, Nagano. Nihon Koshu Eisei Zasshi. 2017;64:25–35. (Japanese).

12. Nagano Prefecture Healthy Longevity Project Study Team. Report on Nagano Prefecture Healthy Longevity Project and Research Program (Analysis of factors in healthy longevity) 2015. https://www.pref.nagano.lg.jp/kenko-fukushi/kenko/kenko/kenkochojupj.html. Accessed 5 Mar 2022.

13. Wood S, Harrison SE, Judd N, Bellis MA, Hughes K, Jones A. The impact of behavioural risk factors on communicable diseases: a systematic review of reviews. BMC Public Health. 2021;21:2110.

14. Ministry of Health, Labour and Welfare, Japan. Summary report of Comprehensive Survey of Living Conditions 2019. https://www.mhlw.go.jp/toukei/saikin/hw/k-tyosa/k-tyosa19/index.html. Accessed 5 Mar 2022. (Japanese).
