# Supplementary material for: Number of public health nurses and COVID-19 incidence rate by variant type: an ecological study of 47 prefectures in Japan
Source: Environ Health Prev Med. 2022 May 3;27:18. doi: 10.1265/ehpm.22-00013 (PMC9251616; doi:10.1265/ehpm.22-00013)
Supplement: Supplementary file 4 — Additional file 4: Association between COVID-19 incidence rate and number of PHNs per population, by variant type: Additional analysis for Model 2. [file ehpm-27-018-s004.pdf]

Additional file 4. Association between COVID-19 incidence rate and number of PHNs per population, by variant type: Additional analysis for Model 2

Number of PHNs	Wild type		Alpha variant		Delta variant		All cases	
per population	IRR (95% CI)	*P*	IRR (95% CI)	*P*	IRR (95% CI)	*P*	IRR (95% CI)	*P*
Model 2 (Original model: proportion of people aged 65 and older)								
5th quintile	1.00		1.00		1.00		1.00	
4th quintile	1.46 (0.93-2.30)	0.099	1.94 (1.13-3.33)	0.016	1.16 (0.86-1.57)	0.324	1.39 (0.96-2.00)	0.081
3rd quintile	1.21 (0.80-1.84)	0.374	1.96 (1.29-2.97)	0.001	1.20 (0.94-1.54)	0.145	1.36 (1.05-1.76)	0.020
2nd quintile	1.42 (0.95-2.13)	0.085	1.83 (1.08-3.09)	0.025	1.31 (1.00-1.71)	0.049	1.41 (1.08-1.85)	0.012
1st quintile	1.84 (1.28-2.65)	0.001	2.35 (1.04-5.28)	0.039	1.55 (1.23-1.96)	<0.001	1.73 (1.28-2.34)	<0.001
P for trend	0.013		0.363		0.003		0.065	
Model 2A (Alternative model: population density)								
5th quintile	1.00		1.00		1.00		1.00	
4th quintile	1.44 (0.89-2.32)	0.139	1.96 (1.16-3.31)	0.012	1.14 (0.78-1.66)	0.503	1.37 (0.91-2.06)	0.135
3rd quintile	1.35 (0.86-2.12)	0.189	1.94 (1.25-3.03)	0.003	1.37 (0.99-1.90)	0.061	1.49 (1.07-2.08)	0.018
2nd quintile	1.61 (1.07-2.42)	0.023	1.79 (1.12-2.86)	0.015	1.47 (1.10-1.98)	0.010	1.56 (1.18-2.06)	0.002
1st quintile	2.01 (1.29-3.14)	0.002	2.24 (1.01-4.98)	0.048	1.65 (1.21-2.26)	0.002	1.82 (1.29-2.59)	0.001
P for trend	0.011		0.270		0.003		0.006	

CI, confidence interval; IRR, incidence rate ratio; PHNs, public health nurses.

Model 2 was adjusted for proportion of people aged 65 and older, proportion of tertiary industry workers, household crowding, and annual mean temperature.

Model 2A was adjusted for log population density, proportion of tertiary industry workers, household crowding, and annual mean temperature.
